# Current Trends in Tolerance Induction in Cow's Milk Allergy: From Passive to Proactive Strategies

**DOI:** 10.3389/fped.2019.00372

**Published:** 2019-09-18

**Authors:** Cansin Sackesen, Derya Ufuk Altintas, Aysen Bingol, Gulbin Bingol, Betul Buyuktiryaki, Esen Demir, Aydan Kansu, Zarife Kuloglu, Zeynep Tamay, Bulent Enis Sekerel

**Affiliations:** ^1^Division of Pediatric Allergy, Department of Pediatrics, Koc University School of Medicine, Istanbul, Turkey; ^2^Division of Pediatric Allergy, Department of Pediatrics, Cukurova University School of Medicine, Adana, Turkey; ^3^Division of Pediatric Allergy and Immunology, Department of Pediatrics, Akdeniz University Faculty of Medicine, Antalya, Turkey; ^4^Division of Pediatric Allergy and Immunology, Department of Pediatrics, Acibadem University School of Medicine, Istanbul, Turkey; ^5^Division of Pediatric Allergy and Asthma, Department of Pediatrics, Hacettepe University Faculty of Medicine, Ankara, Turkey; ^6^Division of Pediatric Allergy, Department of Pediatrics, Ege University School of Medicine, Izmir, Turkey; ^7^Division of Pediatric Gastroenterology, Department of Pediatrics, Ankara University School of Medicine, Ankara, Turkey; ^8^Division of Pediatric Allergy and Immunology, Department of Pediatrics, Istanbul University Istanbul School of Medicine, Istanbul, Turkey

**Keywords:** cow's milk protein allergy, prevention, tolerance, formula, allergenic foods

## Abstract

This review addresses the current strategies of inducing tolerance development in infant and childhood cow's milk protein allergy (CMPA). The change in prevention strategies for CMPA has been emphasized based on the lack of evidence to support the efficacy of food allergen avoidance in infancy and the concept of the dual-allergen-exposure hypothesis, which suggests that allergen exposure through the skin leads to sensitization, whereas early oral consumption of allergenic food protein induces oral tolerance. The new approach is based on the likelihood of early introduction of allergenic foods to the infant's diet to reduce the development of food allergies through oral tolerance induction. The latest treatment guidelines recommend the continuation of breast feeding and the elimination of cow's milk and products from the maternal diet in exclusively breast-fed infants with CMPA, the use of an extensively hydrolyzed infant formula (eHF) with proven efficacy in CMPA as the first elimination diet in formula-fed infants with CMPA and the use of amino acid-based formula (AAF) in severe cases, such as anaphylaxis, enteropathy, eosinophilic esophagitis, and food protein-induced enterocolitis syndrome (FPIES), as well as cases of multiple system involvement, multiple food allergies, and intolerance to extensively hydrolyzed formula (eHF). In conclusion, this paper presents the current knowledge on tolerance development in infants and children with CMPA to increase the awareness of the clinicians concerning the new approaches in CMPA treatment Tolerance development is considered a relatively new concept in CMPA, inducing a shift in interventions in CMPA from a passive (avoidance of responsible allergen) toward a proactive (tolerance induction) strategy.

## Introduction

There has been an alteration in the natural history of food allergy during the previous two decades with an increased prevalence, more severe clinical manifestations and higher risk of persistence into later ages (1–4).

Given that oral exposure is considered to be responsible for allergic sensitization to food, an elimination diet has become the best strategy for the prevention of food allergies, and food allergen avoidance has been the mainstay preventive strategy in food allergy (4–7). However, in addition to the lack of convincing evidence to support the long-term efficacy of food allergen avoidance in the prevention of food allergy, the likelihood of the development of sensitization via allergen exposure through the skin and the induction of tolerance via early oral consumption of allergenic food proteins has also been suggested (4–6).

Accordingly, prior recommendations for the avoidance of peanuts during the first 3 years of life and common food allergens until the first (milk), second (egg), or third (tree nuts and fish) years of life in a diet of an infant at increased risk of atopy by the American Academy of Pediatrics guidelines (7) have been withdrawn and replaced by comments about the lack of current evidence on these topics (8).

Cow's milk protein allergy (CMPA), the most common food allergy in infants and young children (9–13), can induce a diverse range of symptoms involving many different organ systems depending on the type of immune reaction (14). The discrimination of the type of immune reactions is important, since patients with IgE-positive vs. IgE-negative CMPA are considered at increased risk of developing multiple food allergies and atopic diseases, such as asthma, atopic dermatitis, and rhino-conjunctivitis, as well as delayed tolerance (15–18).

Without an appropriate diagnostic workup, there is a risk for both over- and underdiagnosis of CMPA leading to inappropriate elimination diets and thus impaired growth and a poor quality of life (14, 19, 20).

Therefore, this review by experts aimed to provide a guiding document and a comprehensive framework for addressing tolerance development in infants and children with CMPA. The main topics addressed in this paper include (a) an overview of food allergy (prevalence and epidemiology, tolerance development, and risk factors for persistence), (b) CMPA (prevalence, types of immune reaction, clinical presentation, and associated conditions, natural course, and tolerance development), and (c) interventions for allergen avoidance and induction of tolerance (baked milk products, formulas, and immunotherapy) in patients with established CMPA.

## Overview of Food Allergy

### Prevalence and Epidemiology of Food Allergy

According to systematic reviews and meta-analyses of epidemiological studies during the last 2 decades, the frequency of food allergy appears to be increasing in both developed and developing countries, particularly in children (21–25). The prevalence of an oral food challenge (OFC)-proven food allergy is considered to range from 1 to 10% in infants and preschool children (<5 years) and from 0.16 to 2.5% in school-aged children (>5 years) (21).

Food allergy can be induced by IgE-mediated mechanisms, manifesting as immediate urticaria, vomiting, wheezing and anaphylaxis, non-IgE-mediated delayed cell-mediated reactions, or mixed immune reactions to any routes of exposure to culprit foods (3, 22, 26, 27).

The most prevalent food allergens in childhood are cow's milk and eggs, while the third and fourth most common triggers differ depending on the geographical region, age, and dietary patterns, such as peanuts in the United States and Switzerland, wheat in Germany and Japan, tree nuts in Spain, sesame in Israel, walnuts in Korea, and hazelnut in Turkey (3, 10, 28–30).

Food allergy may affect several organs, including the skin, gastrointestinal tract, respiratory tract, and cardiovascular system; moreover, food-induced anaphylaxis is considered to be the most serious and potentially life-threatening reaction, with the highest prevalence in the 0–4 year age group and an increased prevalence in the last two decades, particularly in the 5–14 year age group (3, 26, 27, 31, 32).

### Tolerance Development and Risk Factors for Persistence of Food Allergy

Symptom severity following ingestion, lower reaction eliciting threshold dose, earlier age at diagnosis, and presence and severity of other allergic comorbidities (e.g., eczema, asthma, allergic rhinitis) have been associated with a delayed amelioration of allergy to foods and a higher likelihood of a more persistent food allergy phenotype (33).

Routinely available assays of IgE sensitization include the skin prick test (SPT) and serum food-specific (s) IgE levels. In general, a larger SPT wheal size or higher food sIgE levels are associated with persistent food allergy (33).

Genetic factors, such as atopic family history, male sex, parental ethnicity, atopic dermatitis and related genetic polymorphisms, are important in the development of food allergy (6, 34, 35). Nevertheless, a rapid increase in food allergy prevalence over a short period necessitated evolving strategies to promote allergy prevention through the identification of modifiable environmental factors (i.e., food exposure, the intrauterine environment, and lifestyle factors) (6, 34, 35).

Food allergen avoidance during infancy had limited success in reducing food sensitization and food allergy after the first year of life and has no effect on respiratory allergy or aeroallergen sensitization from birth to age 4 years (35, 36). Moreover, no convincing evidence exists on the benefit of exclusive breast-feeding beyond 4 months of age in preventing atopic disease and in reducing long-term IgE-mediated food allergy in children (6, 35). A recent study showed that cow's milk allergy was less frequent when regular CMP formula was introduced from the first 15 days of life as a complement to breastfeeding than when introduction at an age of 4–6 months (37).

The Learning Early about Peanut Allergy (LEAP) trial showed that the early consumption of peanut compared with avoidance was associated with a significant reduction in the development of peanut allergy by 5 years of age both in high-risk peanut sensitized infants with severe eczema and/or egg allergy (10.6 vs. 35.3%) and in peanut non-sensitized infants (1.9 vs. 13.7%) (38). Thus, early environmental exposure (through the skin) to peanut is considered to account for early sensitization along with the potential contribution of early oral exposure in the development of immune tolerance (5, 38). The Persistence of Oral Tolerance to Peanut (LEAP-On) study showed that the absence of reactivity was maintained in these infants with early consumption of peanut (39).

In the Enquiring about Tolerance (EAT) trial, early introduction of common dietary allergens (peanut, cooked hen's egg, cow's milk, sesame, white fish, and wheat) in small amounts from 3 months of age was studied in exclusively breast-fed infants (*n* = 1,303) compared to infants who were exclusively breastfed until 6 month of age (40). The results showed that early introduction and regular consumption of 2 grams of peanut and egg white protein per week was significantly associated with a lower prevalence of peanut and egg allergy between 1 and 3 years of age compared to infants who were exclusively breastfed for ~6 months (40). However, the early introduction and regular consumption of cow's milk, sesame, white fish, and wheat was not as successful as peanut and egg in this study. We can conclude that randomized controlled trials of oral tolerance induction with early introduction of a group of foods (peanut, egg, milk, sesame, fish, or wheat) obtained variable results. Regarding the limitations of these studies, the meta-analysis by Ierodiakonou et al. (41) reported that early introduction of egg or peanut to the infant diet resulted in a lower risk of developing egg or peanut allergy with moderate certainty; however, the findings for early introduction of milk or hydrolyzed formula were classified as no evidence (42).

Two possible explanations have been proposed for the failure of allergen avoidance in infancy to prevent food allergy. These explanations include the likelihood of sensitization to food allergens to occur through routes of exposure other than oral consumption and the likelihood of the early introduction of some allergenic foods to the infant's diet to reduce the development of food allergies through oral tolerance induction (5, 43).

In this regard, the current concept of the “dual-allergen-exposure hypothesis” suggests that early cutaneous exposure to food protein via disrupted skin barrier results in allergic sensitization, whereas early oral exposure to food allergen induces tolerance (4–6, 40). Low-dose cutaneous exposure to environmental foods (on hands, tabletops, and dust) is considered to penetrate the skin barrier and induce T helper (h) 2 responses and IgE production by B cells. However, early high-dose oral consumption induces tolerance via Th1 and regulatory T-cell responses in the gut-associated lymphoid tissue. The timing and balance of cutaneous and oral exposure are thought to determine whether a child will have an allergy or tolerance (5, 43).

Based on the dual antigen exposure hypothesis, it has been suggested that (a) prompt intensive treatment of eczema in early infancy may decrease inflammation and permeability in the skin and prevent allergic sensitization to foods, (b) reduction in food allergens in the child's environment may lead to a reduction in sensitization, and (c) early introduction of allergenic foods to the infant's diet in small amounts may reduce the development of food allergies through oral tolerance induction (5, 43).

## Cow's Milk Protein Allergy

### Clinical Presentations According to Type of Immune Reaction in CMPA

CMPA is the most common food allergy in infants and young children with a prevalence of 2–7.5%, which accounts for approximately one-fifth of childhood food allergies (1, 9–12, 14, 33, 44).

CMPA is a complex disorder caused by an aberrant inflammatory immune reaction to CMP, classified as “immediate” (up to 2 h after allergen ingestion, typically IgE-mediated) or “late onset” (up to 48 h, typically non-IgE or mixed type) adverse reactions that are distinct from those related to cow's milk intolerances (i.e., lactose intolerance) (9–12, 14, 18, 28, 45–47). CMPA is more prevalent in infants (2–6%) than in adults (0.1– 0.5%), and the disease peaks in the first year of life with a predominance of the IgE-mediated type of allergy (14, 16, 45). According to data from the EuroPrevall birth cohort, the incidences of overall CMPA and IgE-mediated CMPA in the first 2 years of life were reported to be 0.54 and 0.44%, respectively, while the incidence of non-IgE-mediated confirmed CMPA was reported to range from 0.13 to 0.72% across Europe (16).

In most children with CMPA, IgE-mediated CMPA predominates as manifested by generalized systemic reactions (anaphylaxis) or cutaneous, gastrointestinal and/or respiratory reactions along with positive skin tests and/or serum milk sIgE antibodies (28). Disorders involving non-IgE-mediated CMPA only occur in a subset of children and are mainly localized to the gastrointestinal tract, while skin (atopic dermatitis) and rarely respiratory tract reactions (Heiner syndrome) may also occur along with negativity of tests for IgE antibodies (28).

Despite the likelihood of an overlap of clinical symptoms in IgE-mediated and non-IgE-mediated type immune reactions and combinations of immediate and delayed reactions to the same allergen in the same patient, a detailed history and appropriate laboratory studies indicate the correct diagnosis in most cases (14, 28, 48). Distinguishing between the mechanisms of immune reactions is important, as IgE-mediated CMPA is associated with a higher risk of multiple food allergies and atopic conditions, such as asthma, later in life (18, 28, 49).

Overall, the clinical manifestations of IgE-mediated CMPA comprise cutaneous symptoms (70–75%: urticaria, generalized maculopapular rashes, flushing, and angioedema), gastrointestinal symptoms (13–34%: nausea, vomiting, colicky abdominal pain, and diarrhea), respiratory problems (1–8%: nasal pruritus and congestion, rhinorrhea, sneezing, wheezing, dyspnea, chest tightness, and symptoms of asthma and allergic rhinitis), alterations that affect more than one organ system (26%), and severe and potentially life-threatening anaphylaxis (1–4%) (Table 1) (17, 50).

**Table 1:** Clinical presentation of CMPA with respect to type of immune reaction (14, 18, 28).

**Presenting symptoms/signs**	**IgE-mediated CMPA**		**Non-IgE-mediated CMPA**	
Gastrointestinal	Nausea/vomiting Diarrhea Colic Constipation Abdominal pain		Regurgitation Nausea/vomiting Chronic diarrhea Constipation, Colic Blood in stool Food refusal	Dysphagia Dyspepsia Retrosternal pain Malabsorption Failure to thrive or weight loss
Respiratory	Nasal pruritus Nasal congestion Wheezing Cough Rhino-conjunctivitis	Dyspnea Stridor Chest tightness and nasal discharge	Recurrent pulmonary infiltrates associated with tachypnea and recurrent fevers	
Cutaneous	Urticaria Eczema Angioedema Flushing Pruritus		Atopic dermatitis	
**Associated disorders**	**IgE-mediated CMPA**		**Non-IgE-mediated/mixed CMPA**	
	I. Systemic IgE-mediated reactions (Anaphylaxis) A. Immediate-onset reactions B. Late-onset reactions II. IgE-mediated gastrointestinal reactions A Oral allergy syndrome B Immediate gastrointestinal allergy III gE-mediated respiratory reactions A Asthma and rhinitis secondary to ingestion of milk B Asthma and rhinitis secondary to inhalation of milk (e.g., occupational asthma) IV IgE-mediated cutaneous reactions A Immediate-onset reactions 1 Acute urticaria or angioedema 2 Contact urticarial B Late-onset reactions Atopic dermatitis		I Atopic dermatitis A Immediate-onset reactions B Late-onset reactions II Non IgE-mediated gastrointestinal reactions Gastro-esophageal reflux disease (GERD) Crico-pharyngeal spasm Allergic eosinophilic esophagitis (EoE) Severe irritability (colic) Cow's milk-induced iron deficiency anemia Food protein-induced enterocolitis syndrome (FPIES) Food protein-induced allergic proctocolitis (FPIAP) Food protein-induced enteropathy (FPE) III Non-IgE-mediated respiratory reactions Pulmonary hemosiderosis (Heiner syndrome)	

*Adapted from Fiocchi et al. (28), Koletzko et al. (14), and Brill (18)*.

The majority of disorders involving non-IgE-mediated CMPA are localized to the gastrointestinal tract (nausea, vomiting, diarrhea, abdominal pain, blood in stool malabsorption, and failure to thrive or weight loss), and in some cases, atopic dermatitis symptoms may present at the same time (Table 1) (28, 50).

Gastrointestinal symptoms of non-IgE mediated CMPA are characterized by subacute and/or chronic symptoms and may present as a variety of disorders, including cricopharyngeal spasm, gastro-esophageal reflux disease (GERD), allergic eosinophilic esophagitis (EoE), food protein-induced enterocolitis syndrome (FPIES), food protein-induced allergic proctocolitis (FPIAP), food protein-induced enteropathy (FPE), and cow's milk-induced iron deficiency anemia (Table 1) (28, 50, 51).

EoE has become more prevalent over the past decade and is characterized by dysphagia, chest and abdominal pain, food impaction and food refusal, and failure to thrive or weight loss in the more severe cases, which are unresponsive to anti-reflux medications (28).

CMPA is one of the most common causes of FPIES, a form of non-IgE-mediated allergy that develops 1–3 h after the ingestion of milk protein in the acute form and results in repetitive vomiting, hypotonia, pallor, and, in some cases, hypotension and diarrhea (28, 52). Chronic FPIES is an uncommon form that occurs in children with daily consumption of the offending food resulting in chronic or intermittent emesis or reflux, watery diarrhea, and weight loss or failure to thrive (52, 53). FPE is an uncommon disorder that typically presents as diarrhea, failure to thrive, vomiting, and occasionally hypoproteinemia. FPIAP is a relatively benign disorder that results in mild rectal bleeding (i.e., flecks of blood) that may be accompanied with mild diarrhea in an otherwise healthy infant (28).

Heiner's Syndrome is a very rare form of CMPA-related pulmonary hemosiderosis and characterized by recurrent pulmonary infiltrates associated with chronic cough, recurrent fevers, wheezing, rales, tachypnea, and failure to thrive (28).

### Natural Course of CMPA and Development of Tolerance

CMPA has a favorable prognosis with a natural course of onset from the neonatal period, a peak during the first year of life, and remission, with the majority of patients outgrowing the allergy throughout childhood and early adolescence (28, 33).

The reported rates of milk allergy resolution vary by IgE status, genetics, selection criteria, assessment methods, frequency of re-challenge, and study design, while trends toward a delayed resolution of allergy in CMPA and an atopic carrier status in infants who initially recover from CMPA later in life have also been emphasized (1, 28, 33, 43, 54).

The mechanism of the immune reaction in CMPA was shown to be associated with both the rate and timing of tolerance development, with more frequent and earlier development of tolerance in non-IgE mediated CMPA than in IgE-mediated CMPA (16). However, a more favorable prognosis for IgE-mediated CMPA with 65–75% resolution rates until the age of 3–4 years has also been reported in population-based studies (55, 56). On the other hand, the tolerance acquisition in FPIES (non-IgE mediated food allergy) may be delayed when the patient has the co-existence of IgE sensitization to milk (57). Another large population-based cohort study from Israel reported that in patients with CM-induced FPIES, 60% had tolerance by 1 year, 75% by 2 years, and 85% by 3 years (58). Moreover, it is reported that the development of tolerance in cow's milk-induced FPIES occurs earlier than grain-induced and solid food (e.g., egg)-induced FPIES (52).

The levels of sIgE (particularly against sequential epitopes of casein) and antibody binding to other ingestant and inhalant allergens, SPT wheal sizes, severity of eczema at diagnosis, respiratory symptoms with skin and/or gastrointestinal symptoms at onset, persistence of serious symptoms, sensitization to multiple foods, initial sensitization to respiratory allergens and family history of progression to atopic asthma, rhinitis, and eczema were reported to be inversely associated with the timing of CMPA resolution, leading to a higher risk of a longer duration of disease (17, 28, 33, 59). A larger SPT wheal size and/or higher food-sIgE levels at the initial diagnosis were associated with a higher likelihood of persistent food allergy (33). Furthermore, patients with milk sIgE-positive vs. milk sIgE-negative CMPA are at increased risk of developing multiple food allergies as well as atopic diseases, such as asthma, atopic dermatitis, and rhino-conjunctivitis (15).

## Intervention Strategies for Tolerance Induction in CMPA

Overall, the intervention strategies in CMPA have been targeted at three levels: (1) primary prevention of initial IgE sensitization; (2) secondary prevention of the triggering of allergic reactions to interrupt the development of food allergy in IgE-sensitized children; and (3) tertiary prevention to reduce the manifestation of end-organ allergic disease in children with established food allergy via avoidance of allergenic food and induction of tolerance (i.e., baked milk products, formulas, and oral immunotherapy) (6, 45).

For at-risk infants unable to be exclusively breast-fed, the use of hypoallergenic hydrolyzed formulas during the critical risk period for allergic sensitization has been suggested as a preventive strategy (6, 45). However, no evidence exists to support feeding with a hydrolyzed formula for the prevention of allergy compared with exclusive breast-feeding or cow's milk formula (6, 41, 42, 60). No evidence exists either regarding the potential inhibitory role of implementation of an elimination diet or use of supplements (i.e., probiotics) during pregnancy or lactation in development of a food allergy (17). Moreover, in accordance with the dual antigen exposure hypothesis, early consumption of food protein is considered to induce oral tolerance in certain foods (i.e., peanut, egg) (5, 6).

**Figure 1:**
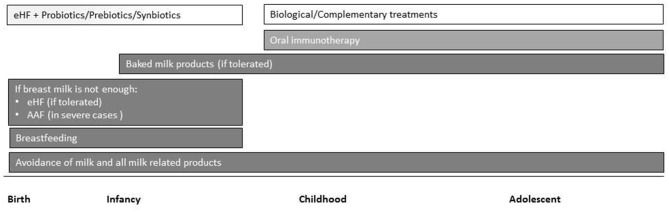
Overview of current and emerging treatment interventions for CMPA in childhood. Intensity of bar color represents the strength of the evidence (eHF, extensively hydrolyzed formula; AAF, amino acid-based formula).

Tertiary prevention in children with established CMPA is based on avoidance of allergenic food and treatments that target tolerance induction. Cow's milk protein exclusion diet (elimination diet) is considered the most effective treatment for CMPA. Maternal breastfeeding is the best strategy with use of extensively hydrolyzed formula (eHF), amino acid-based formula (AAF) or formula that contains soy proteins (after 6 months of age) when breastfeeding is not possible. Recently, implementation of specific oral immunotherapy to achieve an active immune response has been considered in the management of CMPA (Figure 1) (17).

### Avoidance of Allergenic Food

In exclusively breast-fed infants with CMPA, the continuation of breast-feeding along with maternal elimination diet for CMP containing products may be considered in certain cases with the aid of supplemental calcium and vitamin D (46, 61, 62).

In exclusively breast-fed or formula-fed infants with CMPA, weaning food is recommended to be free of CMP until oral challenge tests, dependent on confirmation of the tolerance (14, 61, 62). The introduction of supplementary foods should not be delayed, although it should occur one food at a time in small amounts and only after the infant is at least 17 weeks of age, preferably while the mother is still breastfeeding (14, 63, 64).

### Induction of Tolerance

#### (a) Hydrolyzed Formulas, Probiotics, and Tolerance Development in CMPA

In a previous study of 260 children with CMPA (aged 1–12 months, IgE-mediated CMA in 42.7%), a food challenge performed after 12 months to assess the acquisition of tolerance indicated that an eHCF supplemented with the probiotic *Lactobacillus rhamnosus* GG (LGG) induced higher tolerance rates (78.9%) than eHCF without LGG (43.6%) and other formulas, including AAF (18.2%), hydrolyzed rice formula (32.6%), and soy formula (23.6%) (65). Binary regression analysis indicated a significant association of the mechanism of CMA and the formula type with tolerance development with a higher likelihood of acquiring tolerance in subjects with a non-IgE-mediated mechanism and with the use of eHCF and eHCF + LGG. The authors concluded that eHCF accelerates tolerance acquisition in children with CMPA compared with other dietetic choices, and this effect is augmented by LGG (65).

eHCF with LGG was also shown to reduce the incidence of other allergic manifestations and to accelerate the development of oral tolerance in children with IgE-mediated CMPA (66).

Preliminary data suggested immune-regulatory properties of hydrolyzed casein peptides to be associated with a potential long-term effect of dietary intervention with eHCF +LGG on the immune system and tolerance induction via positive effects on gut dysbiosis, short-chain fatty acid production, and epigenetic regulation of Th1 and Th2 cytokine gene expressions (67–69). However, given that the effect of eHF with LGG on tolerance acquisition has been based on a limited number of studies, further RCTs investigating the effect of eHF with pre-probiotics are needed to clarify its role in the development of tolerance in CMA.

#### (b) Modified Formula

In formula-fed infants, the first elimination diet is an eHF with proven efficacy and cost effectiveness in CMPA (14, 61, 70, 71). An AAF may be considered as the first choice in infants with severe or life-threatening symptoms (Table 2) (14, 61).

**Table 2:** Choice of formula in non–breast-fed infants with confirmed CMPA according to clinical presentation (14, 17, 52, 61).

**CMPA Clinical presentation**	**1st choice**	**2nd choice**	**Key points**
Anaphylaxis	AAF	eHF	
Immediate gastrointestinal allergy	eHF	AAF	Differential diagnosis of anaphylaxis should be made
Food protein-induced enterocolitis syndrome (FPIES)	AAF/eHF		AAF in previous guidelines, whereas extensively hydrolyzed casein formula (eHCF) in International FPIES (2017) guidelines is recommended as the first choice. A case-based evaluation is necessary until sufficient clinical experience is available. AAF should be considered the first option in FPIES patients with hypoalbuminemia
Atopic dermatitis	eHF	AAF/SF in infants aged > 6 months	AAF should be considered the first choice in breast feeding infants with CMPA or severe atopic dermatitis.
Allergic eosinophilic esophagitis	AAF		
Food protein-induced enteropathy	eHF	AAF	AAF should be considered the first choice in enteropathies accompanied with hypoproteinemia.
Food protein-induced allergic proctocolitis	eHF	AAF	
Milk-induced chronic pulmonary disease (Heiner's syndrome)	AAF	SF in infants aged > 6 months	
Asthma and rhinitis	eHF	AAF/SF in infants aged > 6 months	Anaphylaxis should be ruled out via differential diagnosis May be considered in treatment failure for asthma/rhinitis Solitary presence is rare and often involves other systems
Acute urticaria or angioedema	eHF	AAF/SF in infants aged > 6 months	Anaphylaxis should be ruled out via differential diagnosis
Gastroesophageal reflux disease (GERD)	eHF	AAF	Frequent in infancy. In cases with failure to classical treatment, diagnosis of CMPA should be considered
Constipation	eHF	AAF	Frequent in infancy. In cases with failure to classical treatment, diagnosis of CMPA should be considered
Infantile colic	eHF	AAF	Frequent in infancy. In severe cases, CMPA should be considered in the differential diagnosis.

eHF, extensively hydrolyzed formula; AAF, amino acid-based formula; SF, soya formula.

*Adapted from Koletzko et al. (14), Martorell-Aragonés et al. (17), Kansu et al. (61), and Nowak-Wegrzyn et al. (52)*.

The risk of reaction or no response to eHF is far below 10% of the infants with uncomplicated CMPA and is more common, reaching up to 40% in patients with a more severe syndrome and multiple food allergies (17, 72–77). AAF provides a safe alternative for individuals who are allergic to eHFs and provides the ability to refine the diagnosis of eHF allergy (72, 73, 78).

Soy protein formula, if tolerated, is an option beyond 6 months of age, whereas it is not indicated in situations of enteropathy or non-IgE-mediated allergies that are sensitive to soy (Table 2) (14, 17).

In a systematic review on the efficacy of AAF in relieving the symptoms in CMPA, the analysis has demonstrated that for infants with CMPA who completely tolerate eHF, there was no additional benefit from the use of AAF (79). The authors noted that AAF and eHF were equally effective in resolving gastrointestinal and skin symptoms in uncomplicated CMPA based on evidence from head-to-head randomized controlled trials (RCTs), whereas a clear need for AAF was shown in infants with intolerance to eHF (79). Given that eHF-intolerant patients were also more likely to have severe atopic eczema, anaphylaxis, reflux esophagitis or FPIES and FPIAP with failure to thrive, more severe symptomatology is considered necessary to warrant the use of AAF (4, 6, 73, 75, 79–81).

In patients with CMPA, eHF may be the primary choice when the clinical presentation includes colic, constipation, GERD, acute urticaria, acute angioedema, atopic dermatitis, and gastroenteritis, whereas AAF may be the primary choice in severe cases, such as anaphylaxis, enteropathy, eosinophilic esophagitis and FPIES, as well as cases of multiple system involvement, multiple food allergies, and intolerance to eHF (Table 2) (7, 14, 28, 35, 74, 82).

The specific immunologic mechanisms that drive most of the non-IgE-mediated food allergies are largely unknown; therefore, the therapies for these conditions remain non-specific (53). In FPIES, breastfeeding can be continued unless maternal ingestion of a non-identified allergen triggers acute or chronic-FPIES. An eHF is usually well-tolerated, although up to 20% of patients may need an AAF (51). AAF formula was the primary choice in previous guidelines, whereas extensively hydrolyzed casein formula (eHCF) in International FPIES (2017) guidelines is recommended as the first choice. AAF should be considered the first option in FPIES patients with hypoalbuminemia (52). Infants with milk/soy-FPIES may be breastfed, and if the severity of FPIES is mild to moderate, a hypoallergenic formula approved for infants with milk allergy, such as eHCF, may be used (52). A case-based evaluation is necessary until sufficient clinical experience is available.

In FPIAP, elimination of food from the maternal diet is usually sufficient. In rare cases, the identification of the causative factor may be difficult. An eHF or AAF might be necessary if breastfeeding is not an option or if blood in stools becomes severe (Table 2) (51).

#### (c) Baked-Milk Products

IgE-antibody production is primarily directed at heat-sensitive conformational epitopes in transient milk allergy, whereas IgE antibodies are also produced against heat-stable sequential epitopes in persistent allergy. Greater IgE-epitope diversity and higher IgE affinity are related to more severe milk allergy (83).

Given the reduction in allergenicity by destruction of conformational epitopes of milk proteins via extensive heating or food processing, children with transient milk allergy are considered likely to tolerate baked-milk products (62, 83). Accordingly, clinical trials indicated that nearly 75% of children with IgE-mediated CMPA tolerate baked milk-containing foods, such as muffins, cakes and bread, and the inclusion of baked milk products in the children's diet is suggested to accelerate the development of unheated-milk tolerance compared to a strict milk avoidance approach, which is currently the standard of care (83–86). Tolerance to baked milk is a marker of transient IgE-mediated cow's milk allergy, whereas reactivity to baked milk portends a more persistent and severe phenotype of CMPA with a higher risk of severe anaphylaxis and a more protracted course (83, 87). The addition of baked-milk to the diet appears to accelerate the development of unheated-milk tolerance compared to strict avoidance, along with a significant increase in casein IgG4 values in the baked-milk-tolerant group, similar to those spontaneously outgrowing milk allergy and treated with milk oral immunotherapy (OIT) (83, 88–92). Recent studies on the regular consumption of baked milk and related immunologic changes reinforce baked milk as a proactive treatment for food allergy (85, 86, 93–95). A randomized controlled trial assessed the effect of baked milk on accelerating tolerance in 84 children with CMPA who tolerated baked milk in oral food challenge (OFC) (96). The tolerance rate of the children in the case group who consumed baked milk products for 1 year was higher than that in the avoidance group (88.1 vs. 66.7%, p:0.018). While the introduction of baked milk into the diet was demonstrated to accelerate tolerance, the initial sIgE levels of milk, casein, and beta-lactoglobulin did not predict the tolerance of unheated cow's milk (96).

Although the ingestion of baked-milk products is considered a form of immunotherapy with more favorable safety, higher convenience, lower cost, and less labor intensity when compared to OIT, a clear need for strict avoidance in a subset of milk-allergic patients is emphasized, since nearly 25% of children were initially baked-milk-reactive. In addition, the likelihood of treatment discontinuation due to reactions to lesser-cooked forms of milk in nearly 10% of children who passed the initial muffin challenge is considered to highlight the challenges of strict adherence to proper food preparation (83).

The consumption of baked milk is suggested to enhance the quality of life by removing unnecessary dietary restrictions and to change the natural evolution of milk allergy by promoting the development of tolerance to regular cow's milk (83, 85–87, 94).

The “milk ladder” classifies factors associated with the allergic potential of cow's milk food stuffs in terms of volume or quantity, effect of heating and wheat matrix effect from Stage 1 (small quantity, baked and matrix) to Stage 4 (fresh milk products) (Table 3). The use of the milk ladder in patients who can tolerate baked milk may facilitate the introduction of less allergic milk products (97, 98).

**Table 3:** Milk ladder: classification of cow's-milk-containing foods^*^ (97, 98).

**Stage 1** Baked products (at least 180^°^C heating and 30-min duration) containing cow's milk protein: - Small crumb of a biscuit containing < 1 g of protein per biscuit - Build up to 1 biscuit over 5 weeks as tolerated.	**Stage 2** Other baked products containing cow's milk protein: - Biscuits, - Cakes, - Muffin, - Pie, Butter. Margarine.	**Stage 3** Products containing cooked cheese or whole cow's milk as a heated ingredient: - Custard, - Cheese sauce, - Pizza, - Rice pudding, Chocolate. Chocolate-coated items. Fermented desserts, Yogurt, Fromage frais.	**Stage 4** Uncooked cheese Uncooked non-yogurt desserts, for example, ice cream or mousse. Cow's milk UHT milk followed by pasteurized milk and then unpasteurized milk (if this form is preferred by the family).

Stage 1: Small quantity, baked, and matrix. Stage 2: Larger quantity, baked and matrix OR traces without matrix, or with minimal heating. Stage 3: Larger quantity, less heating, and less matrix OR all with some degree of protein change with heating or manufacturing. Stage 4: Fresh milk products.

At all stages, start with a small amount, and gradually increase. Each individual product in Stage 3 is to be initially introduced in trace amounts, as they have more milk protein and a lower degree of heat treatment or protein denaturation. There is also variability in milk protein between products. If a reaction occurs, the food that caused the reaction should be stopped, and reintroduction should be continued with food from a lower stage in smaller amounts.

* *It is more appropriate to use the milk ladder in non-IgE mediated CMPA; it is not advisable in infants/children with prior anaphylaxis to small amounts of milk, asthma, or very high cow's milk sIgE or large skin prick test wheals*.

#### (d) Oral Immunotherapy

OIT has been applied in treating food allergy for over a century and involves monitored repeated administration of gradually increasing doses of allergen over months to years to enable non-reactivity to foods (desensitization) (22, 83, 91, 92).

The data from meta-analyses indicated a ten times greater probability of tolerance development with OIT compared to a strict elimination diet in patients with IgE-mediated CMPA (99). In addition, a significant reduction in skin test positivity to the food allergen, an increase in the specific IgG4 titers, and a substantially lower risk of reactions to the allergen have been shown among individuals administered OIT (17, 100).

Although OIT may be effective in raising the threshold of reactivity to a range of foods in patients with IgE-mediated food allergy while receiving (desensitization) and post-discontinuation of OIT, it was also associated with an increased risk of local and systemic adverse events (22, 101).

The meta-analysis of the Cochrane Collaboration concluded that OIT is effective for inducing desensitization in most patients with IgE-mediated CMPA, whereas albeit mild and self-limiting in most cases, adverse effects are frequently observed, thus limiting its use by selected patients over 3 years of age and in centers with experience in the management of OIT and the capacity to deal with the possible adverse reactions (17, 22, 102).

The most common adverse events related to OIT are observed in the gastrointestinal system, and EoE is of particular concern because of the significant association with OIT. The meta-analysis conducted by Petroni and Spergel determined the incidence of EoE in patients with OIT for milk, egg and peanut (103). The incidence of OIT-related EoE was 5.3% of patients receiving food OIT in the studies that showed the diagnosis of EoE by biopsy findings (104). This observation suggests that food OIT could increase the risk of EoE development, and additional studies investigating the long-term effect of OIT with standard diagnostic procedures for EoE are needed.

## Conclusion

In conclusion, this review by experts from Turkey aimed to document the current knowledge on tolerance development in infants and children with CMPA to increase the awareness of clinicians concerning the new approaches in CMPA treatment, given the change in the natural history and prevalence of food allergy during the last two decades and the related changes in the guidelines in terms of prevention and tolerance induction strategies in food allergies in recent years. Accordingly, the change in prevention strategies for food allergy has been emphasized in this paper. This change in prevention strategies is based on the lack of evidence to support the efficacy of food allergen avoidance in infancy and the concept of the dual-allergen-exposure hypothesis, which suggests that allergen exposure through the skin leads to sensitization, whereas the early oral consumption of allergenic food protein induces oral tolerance. The early introduction of allergenic foods to the infant's diet in small amounts is considered likely to reduce the development of food allergies through oral tolerance induction.

In exclusively breast-fed infants, continuation of breast-feeding is recommended with elimination of cow's milk and products from the maternal diet when an infant reacts to the amount of milk protein passed on from maternal consumption during breastfeeding. If breastmilk is not sufficient, the use of supplemental formula should be considered in CMPA. No delay in the introduction of complementary feeding in infants with CMPA is recommended. Nearly 75% of children with IgE-mediated CMPA tolerate baked-milk-containing foods; thus, the consumption of baked milk could change the natural evolution of milk allergy by promoting the development of tolerance to regular cow's milk and enhancing the quality of life by removing unnecessary dietary restrictions.

The risk of reaction or no response to eHF is far below 10% of infants with uncomplicated CMPA and is as common as 40% in those with a more severe syndrome and multiple food allergies. Accordingly, if a supplemental formula is required, an eHF or AAF may be utilized. This decision must be made on an individualized basis, with use of an eHF with proven efficacy in CMPA as the first elimination diet in formula-fed infants with CMPA. However, AAF should be used in severe cases, such as anaphylaxis, enteropathy, EoE and FPIES, as well as cases of multiple system involvement, multiple food allergies, severe atopic dermatitis, and intolerance to eHF. The use of eHF with the supplementation of the probiotic LGG shows promising results on the early acquisition of tolerance in mild cases of cow's milk allergy. Additional studies investigating the effect of eHF with pre-probiotics will clarify its role in the development of tolerance in CMA. Although OIT is effective in raising the threshold of reactivity to a range of foods in patients with IgE-mediated food allergy, the use of OIT should be restricted to selected patients over 3 years of age and in a center with experience in the management of OIT, given the increased risk of local and systemic adverse events.

Finally, the tolerance development seems to be a relatively new concept in CMPA, inducing a shift in the treatment of CMPA from a passive (avoidance of responsible allergen) toward a proactive (tolerance induction) strategy. However, it should also be kept in mind that currently there is no evidence-based protocol for the strategy of tolerance in most children with CMPA, and further studies are needed. Given the recently described different clinical phenotypes of food allergy, it seems necessary to adopt an individualized nutrition and treatment algorithm that is tailored to each individual's needs and medical conditions in the management of CMPA.

## Author Contributions

CS had primary responsibility for the manuscript preparation. All authors have read and approved the final manuscript.

### Conflict of Interest Statement

The authors declare that the research was conducted in the absence of any commercial or financial relationships that could be construed as a potential conflict of interest.

## Abbreviations

AAF: amino acid-based formula
CMP: Cow's Milk Protein
CMPA: Cow's Milk Protein Allergy
EAT: Enquiring about Tolerance Trial
eHF: extensively Hydrolyzed Formula
EoE: Eosinophilic esophagitis
FPIES: Food protein-induced enterocolitis syndrome
LEAP: Learning Early about Peanut Allergy
OFC: Oral Food Challenge
OIT: Oral Immunotherapy
RCT: Randomized Controlled Trials
sIgE: specific Immunoglobulin E
SPT: Skin Prick Test.
